# Mental Health Related Stigma as a ‘Wicked Problem’: The Need to Address Stigma and Consider the Consequences

**DOI:** 10.3390/ijerph15061158

**Published:** 2018-06-02

**Authors:** Claire Henderson, Petra C. Gronholm

**Affiliations:** 1 Health Service and Population Research Department, Institute of Psychiatry, Psychology & Neuroscience, King’s College London, De Crespigny Park, London SE5 8AF, UK; p.c.gronholm@lse.ac.uk; 2 Personal Social Services Research Unit, London School of Economics and Political Science, Houghton Street, London WC2A 2AE, UK

**Keywords:** stigma, discrimination, intergroup contact, wicked problems

## Abstract

Recent reviews on the evidence base for mental health related stigma reduction show that under certain conditions interpersonal contact is effective in promoting more positive attitudes, reduced desire for social distance, and increased stigma related knowledge (knowledge which disconfirms beliefs based on stereotypes). Short-term interventions may have effects that are attenuated over time; longer term programmes may support sustained improvements, but research following up long-term interventions is scarce. However, the effectiveness of these interventions should not obscure the nature of stigma as a social problem. In this article we describe stigma as a ‘wicked problem’ to highlight some implications for intervening against stigma and evaluating these efforts. These include the risks of unintended consequences and the need to continually reformulate the concept of stigma, to ensure that tackling stigma at the structural, interpersonal, and intrapersonal levels become part of the core business of stakeholder organisations. We compare the main targets of anti-stigma programmes with what is known about the sources of stigma and discrimination and their impacts to identify targets for future intervention. In some cases, interventions have been directed at the interpersonal level when structural level intervention is also needed; in others, systematic reviews have not so far identified any interventions.

## 1. Introduction

While the impact of short-term anti-stigma interventions may be short-lived [1,2,3], the long-term outcomes of current programmes lasting years cannot yet be examined. Their long-term impact may depend in part on legacy activities, that is, activities that organisations funded to deliver anti-stigma programmes for a defined period hand over to other organisations, or help other organisations to initiate. The evidence base and sustainability of these activities are therefore important features on which the current programmes should be judged.

In light of this uncertainty about whether current anti-stigma interventions can have a lasting impact, this article aims to place the current evidence base on interventions in the context of the evidence on the impacts of stigma and discrimination, to show how further work can be best aligned to the sources of discrimination and the levels at which they operate. As a starting point, we discuss the concept of mental health related stigma as a ‘wicked problem’, as described by Rittel and Webber in 1973 [4]. Wicked problems are complex and difficult to define; as their formulation depends on what solutions have been conceived of to tackle them, definitive formulation is not possible. Thus, while it would be wrong to assume that, as a complex and universal problem, stigma is somehow intractable, it is also wrong to approach stigma as a ‘tame’ problem, with an easily agreed definition and technical solutions, or as a battle which can be won or lost [5]. In light of this, we identify some implications for researching stigma, intervening against it, and conducting evaluative research on anti-stigma interventions.

## 2. Stigma as a Wicked Problem

The stigma and discrimination associated with mental health constitute a worldwide multifaceted problem. Rittel and Webber pointed out that every specification of a social problem also contains the direction in which it may be tackled. The nature and hence solutions to problems such as stigma are open to interpretation, so consensus on one definition (and set of solutions) is not an appropriate aim. Instead, the field of stigma research contains a number of definitions, targets, and outcomes.

Link et al.’s sociological perspective on stigmatisation [6] describes it as a process beginning with labelling; on the basis of this label follows stereotyping, separation, emotional reactions, status loss, and discrimination, which can only occur in the presence of a power differential between those labelled and those who label. A definition more rooted in social psychology emphasises stereotypes, prejudice, and discrimination [7], while one oriented for anti-stigma programmes as public health interventions defines stigma as problems of knowledge (ignorance or misinformation), attitudes (prejudice), and behaviour (discrimination) [8]. A summary by Pescosolido and Martin [9] outlines how definitions can be considered to reflect either an experiential or action-orientated perspective for categorising stigma. The former considers stigma in terms of whether it is perceived (a belief “most people” are considered to hold), endorsed (expressing agreement with stereotypes/prejudice/discrimination), anticipated (expecting an experience of prejudice/discrimination), received (overt experiences of rejection or devaluation), or enacted (exhibiting discriminatory behaviours). The latter perspective captures what or who gives or receives the stigma, in terms of public stigma (stereotypes, prejudice, and discrimination as endorsed by the general population), structural stigma (prejudice and discrimination through laws, policies, and constitutional practices), courtesy stigma (stereotypes, prejudice, and discrimination acquired through a connection with a stigmatised group/person), provider-based stigma (prejudice and discrimination by occupational groups designated to provide assistance to stigmatised groups), and self-stigma (when people who belong to a stigmatised group legitimise publicly held stereotypes and prejudice, and internalise these by applying them to themselves).

Although a research focus on defining and understanding stigma has been criticised as being at the expense of intervention research [8], one implication of conceiving of stigma as a wicked problem is that these two types of research should not be set up in opposition to each other. While research designs may differ, they have essentially the same aim; that of reducing stigma.

Following Rittel and Webber’s first point (of ten) of there being no definitive formulation of a wicked problem, as a second point they note that there is no stopping rule for wicked problems. People stop working on them for reasons other than their solution, such as lack of funding or a decision that ‘enough’ has been done. Presenting stigma as a ‘tame’ and finite problem amenable to a single or brief intervention will therefore lead to disappointment, particularly regarding the medium to long-term effectiveness of such interventions. A potential adverse consequence of this disappointment is reduction in funding to address stigma. Interventions such as contact-based education, which applies intergroup contact theory to reduce prejudice [10], can be powerful in the short term [11,12]. However, if applied briefly the effects may wear off, perhaps due to the preceding and successive effects of public stigma. These include implicit attitudes learned at an early age [13,14], and continued exposure to sources of stigma after a brief intervention, for example negative media stereotypes [15], others’ attitudes, or discriminatory policies. Improvements in the application of intergroup contact theory and in the evaluation of interventions based on this theory are therefore needed.

Rittel and Webber’s third point about wicked problems is that instead of solutions being true or false, they tend to be judged as being good or bad. While it is possible to show that an anti-stigma intervention is effective in relation to chosen measures, both the nature of the intervention and the evaluation design are open to value judgements. For example, it may be hard to achieve consensus regarding the targets for an anti-stigma intervention, and the evaluation design can be criticised in terms of whether the measures are valid or more broadly in terms of, for example, its positivist approach. Unintended consequences of solutions to wicked problems are highly likely; this also makes them vulnerable to criticism. For example, protest as a way to address stigma may be counterproductive [16]. Further, methods which are generally effective may be counterproductive for population subgroups; contact may not work as well for youth [16] and for some youth it may be detrimental [17]. Another concern is that a focus on common mental disorder such as that taken by the anti-stigma programme BeyondBlue in Australia [18] may not have any impact on attitudes toward people with schizophrenia; it might even exacerbate stigma against this group. At the level of self or internalised stigma, interventions taking a therapeutic approach such as psychoeducation [19], narrative enhancement and cognitive therapy [20], and cognitive therapy [21] have shown positive effects on at least some outcomes, if not always on internalised stigma measures [22]. However, they have been criticised for pathologising internalised stigma as something requiring treatment instead of it being a predictable consequence of public stigma and experienced discrimination [23]. Alternative interventions focus more on social contact, both through peer contact and intergroup contact [24,25].

The explicit or implicit messages about the nature of mental illness contained in anti-stigma programme content [26] can also lead to unintended consequences. Biological explanations may increase the desire for social distance [27,28], while messages that stress the high incidence of mental health problems may lead to reconceptualization of responses such as grief or stress as mental illness [29]. While it is possible to simply omit some messages to avoid such consequences, an important developmental step in any campaign is to review the evidence on different messages, such as a continuum [30] or biopsychosocial explanation as an alternative to a biological one. This may then be followed by pilot testing in limited samples of different types of messages and explanations, using focus groups and localised campaigns [31].

These unintended consequences contribute to the broad, undefinable and hence unmeasurable set of consequences of anti-stigma interventions as solutions to wicked problems. In common with the evaluation of other complex interventions, the evaluation of an anti-stigma intervention using a clearly defined and accessible sample can use a mixed methods approach so that qualitative work can identify unpredicted consequences. This is impossible at the level of a national campaign, although the use of social media makes it easier to identify consequences at the anecdotal level. Another problem affecting national campaigns is the difficulty of attributing observed changes to the campaign, given the possible influence of other societal changes. To try to address this, comparisons with other countries without such campaigns can be made [27,32]; extrapolation of existing trends can be compared with actual changes observed [33]; and the relationship between campaign awareness and the outcomes can be examined [34]. These means have been used to provide supportive evidence, albeit indirect, in the case of the Time to Change anti-stigma programme in England [33,34].

Just as the potential consequences of an anti-stigma intervention cannot be fully defined or measured, the potential solutions to stigma are not possible to formulate in any complete way. Some solutions are likely to overlap with solutions to other problems, such as the stigma of unemployment and other forms of discrimination which intersect with mental health related stigma. Further, among the potential solutions, it is not possible to appraise these ahead of time in order to identify those most likely be effective. As each wicked problem is unique, an intervention which appears to work for a problem cannot be assumed to work as well for another similar one. Thus, despite evidence on the effectiveness of intergroup contact in reducing stigma, intergroup contact interventions designed to reduce conflict between ethnic or religious groups may require adaptation to be effective for mental health related stigma [11].

## 3. The Impacts of Stigma and Discrimination

One way to describe the complexity and breadth of impact of stigma as a wicked problem and identify areas for intervention is to consider the range of life areas in which discrimination is apparent. Within their communities, people with mental illness often face public stigma in terms of derogatory attitudes and beliefs [35,36]. The preference for social distance from people with mental illness leads to social isolation, loneliness, impaired social networks [37], and reduced social capital [38] and community participation. This particularly affects people living with severe mental illness [39,40]. At the same time, people with severe mental illness are at risk of targeted violence and hostility, such as verbal and physical abuse, and sexual and financial exploitation [41].

The impact of stigma within communities can also be considered in terms of its specific influence within key domains like employment, education, housing, the criminal just system, health and social care, and welfare benefits [42].

Regarding the employment setting, research indicates that stigma can influence the extent to which people with mental illness can obtain and retain jobs [35,43]. For example, a systematic review found that employers generally rated people with mental health problems less employable than people with physical disabilities or no disabilities [44], and a small-scale experimental study found that applicants who disclosed a history of mental illness received fewer call-backs to job interviews (Hipes et al., 2016). The effects of stigma and discrimination in the employment setting appear to be particularly pertinent for people with mental illness at times of general economic hardship [45,46]. Once in employment, co-workers’ negative attitudes are reported to affect how people with mental illness are treated. For example, they may be looked down on or treated as less intelligent [47], or have fewer opportunities for career advancement [48]. Stigma can also impact employment prospects through people with mental illness stopping themselves from looking or applying for jobs, due to anticipated discrimination [49].

In the education setting, students with mental illness or symptoms indicative of such problems can face adverse reactions from peers, such as social exclusion, loss of friendships, teasing, and loneliness [50,51]. Systematic reviews on mental health difficulties amongst university students report that stigma is a reported key barrier to seeking help, which can hinder or contribute to concerns regarding academic and career progression [52,53]. Stigma and discrimination can also result in the exclusion of people with mental illness from higher education [54,55].

Within the housing setting there is evidence of the impact of stigma in the leasing of unsafe housing to people with mental illness [35]. Evidence indicates that tenants with mental illness are often at disadvantage in the housing rental market, and often end up in substandard accommodation (e.g., issues with overcrowding, noise levels, and neighbourhood desirability) [56]**.** Ultimately, inappropriate housing together with other problems such as poor access to employment, welfare benefits and mental health care and substance misuse treatment lead to an increased risk of homelessness amongst people with mental illness [57].

Discrimination within social care is a less studied problem than that within some other public services. However, the experiences of parents with mental illness [58] suggest that the approach taken by social services for families may be less supportive than for parents with other disabilities.

Contact with the police is another reported key domain of discrimination and unfair treatment amongst people with mental illness [42,59]. Contacts with the police can be relatively common for people with mental illness, as members of the police force are frequently involved in facilitating pathways to mental health care [60]. Contact with the police can also occur by virtue of being a victim of crime, as people with mental illness have a higher risk of experiencing violent and non-violent victimisation [61,62]. In this setting, stigma can manifest, for example, in reports of being the victim of a crime being discredited and disbelieved [63].

People with mental illness also report experiences of discrimination in relation to the welfare benefits system [1,64]. Evidence from the UK contexts suggests this might be, for example, due to the procedures through which eligibility for benefit support is assessed, and also attitudes from staff [64].

The stigma associated with mental illness and mental health services and associated anticipated concerns is frequently reported as a reason for people not seeking help or for not fully engaging with available support, thus constituting a barrier to care [65,66]. However, when health care is sought, it may also be experienced as a source of discrimination due to the behaviour of healthcare providers [67]. Stigma amongst healthcare providers can also result in people with mental illness receiving unequal attention and substandard care for physical health complaints [68]. Within mental health services, provider stigma has been shown to be associated with disempowerment among service users, with self-stigma partially mediating this relationship [69]. This impact of stigma has also been reported by health care staff who themselves experience mental health problems [70]**.**

## 4. Anti-Stigma Interventions: Targets, Methods, and Evidence

Having framed stigma as a wicked problem and described the main life areas where it has an impact, we now turn to intervention. To match interventions to the nature and scope of the problem requires sustained work across these life areas. Anti-stigma interventions identified in recent systematic reviews reflect the range of life areas and sectors described above to a partial extent. Reflecting the importance of reducing stigma to try to increase community participation and social capital, many anti-stigma efforts have targeted the general public, whether at national or regional level. In terms of evaluated efforts, improved outcomes on a range of measures (e.g., attitudes, knowledge/mental health literacy, overall discrimination, intended social contact) have been reported, for example, in relation to the BeyondBlue depression-specific programme in Australia [71], the Like Minds, Like Mine programme in New Zealand [72], See Me in Scotland [32], Hjärnkoll in Sweden [3], Opening Minds in Canada [17,73], and Time to Change in England [1,2,33].

In terms of approaches underpinning anti-stigma efforts, these have been classified as reflecting strategies of education (challenging and rectifying inaccurate myths about mental illness), contact (facilitating direct or in-direct interactions with a person with mental illness), and protest (objecting to and suppressing negative representations of mental illness) [74]. Early anti-stigma efforts often used educational approaches [75,76]. There is, however, growing evidence of the effectiveness of intergroup contact in challenging stigma [16], and contact-based interventions have become more common in recent years. The contact provided, through delivery or co-delivery by people with lived experience of a mental health problem, may be face to face or remote, for example, through the use of filmed testimonies or interviews. Such interventions commonly include an educational component to improve mental health literacy, tailored to the target group. While there is evidence for improved knowledge as a mediator of the effectiveness of contact interventions, research on intergroup contact to reduce inter-ethnic prejudice suggests that the reduction of anxiety about having such contact and the promotion of empathy may be more important mediators [77]. The relative importance of these and other possible mediators is underexplored in interventions to reduce mental health related stigma, and may also vary depending on the target group for stigma reduction [78]. In addition to neglect of mediators, the literature on contact interventions rarely provides information on important aspects of implementation such as dose, reach, and fidelity. As it seems that interventions’ effects are often attenuated over time, their sustainability in any given setting is a further question to which almost no attention has been given.

Healthcare professionals form one target group for stigma reduction. While their clinical knowledge and frequent contact with individuals with mental illness might as first glance make contact-based education appear redundant, an understanding of what makes these interventions effective shows how important they are for health professionals. Equalisation of status between groups, disconfirmation of stereotypes, acquaintanceship potential, and collaboration are important facilitators of the effects of intergroup contact [79]. These are usually lacking in acute clinical encounters with people who are severely ill; however, the more positive attitudes among mental health professionals with longer experience [80] suggests that aspects of clinical practice may to some extent be corrective, for example the long-term relationships in community-based mental health care and primary care. Reflective of the relevance of stigma reduction for health professionals, a review found that attitudinal outcomes generally improved following primarily education-based interventions to reduce stigma amongst various groups of health and mental health professionals [80]. Some studies also reported improved outcomes related to knowledge, behaviour, and clinical competence. Follow-up assessments were uncommon, but when available evidence indicated that positive changes were sustained [81,82]. Recent work has also identified the key ingredients associated with attitude change following contact-based stigma reduction education programmes (e.g., social contact in form of personal testimonies, multiple forms of social contact, focus on behaviour change, emphasising recovery) [73], to inform face-to-face stigma-reduction training for healthcare providers in the Canadian Opening Minds programme [83].

Student populations constitute another key target group, as reducing stigma amongst the younger population could help foster new generations with more tolerant and less discriminatory views. A systematic review examined the effectiveness of anti-stigma interventions targeting student groups [84]. Interventions used a variety of approaches, including contact with a person with mental illness, and education (via written, taught or video materials, or role play). Contact-based interventions (whether live or video) were most effective in reducing desire for social distance and improving attitudes.

Criminal justice professionals represent another important target for anti-stigma efforts, given their position of power and the inevitably biased nature of their professional contact experiences with people with mental illness, which seem likely to confirm stereotypes such as that of dangerousness or odd behaviour. There are, however, rather few reports of interventions focusing on stigma reduction amongst this group [85]. Following a training intervention with one police force in England, improvements in attitudes were reported, but there were no changes in perceived knowledge or in challenging the stereotyped view that people with mental health problems are violent [86]. Also, at follow-up, many participants reported improvements in police work (improved communication between police officers and people with mental illness). Likewise, police officers’ attitudes, mental health literacy, and intentional beliefs were improved following a police officer training programme in Sweden, and these improvements generally persisted at 6-month follow-up [60].

A recent systematic review identified a few studies of interventions with journalists with potentially promising results [87]. Interventions reported in the literature for employers focus on mental health literacy and have had positive results on stigma outcomes [88]; other interventions by anti-stigma programmes have included reviews of workplace policies [89] and identification of mental health champions in workplaces [90], but these have not been subject to academic evaluation. No reports of studies have been found targeting other important sectors such as housing, social care, and welfare benefits. If any of these sectors are the targets of anti-stigma programmes, academic evaluation has been lacking.

In addition to the absence of some important targets, there is also at times a mismatch between the levels at which stigma operates and at which anti-stigma programmes intervene. The interventions summarised above mainly target stigma at the interpersonal level. However, as outlined earlier, stigma has been formulated as a problem operating at multiple levels: structural discrimination in terms of legislation and policy; interpersonal stigma, manifested through for example stereotype endorsement, desire for social distance, and discriminatory behaviour; and at the intrapersonal level, self- or internalised stigma, or the internalisation of negative stereotypes of mental illness and consequential damage to self-concept and self-esteem. An example of this mismatch is that of health care. Health care professionals are increasingly a target for contact-based education interventions; while this approach can be effective at least in the short term, it does not address the systemic context in which they work. Some authors have indeed identified health care provider stigma as a form of structural discrimination [91]. The same argument applies to the discrimination experienced within multiple life areas when in contact with public sector services and employers. In their review of intergroup contact, Al Ramiah and Hewstone [10] caution against the neglect of structural discrimination as an unintended consequence of focussing on the effectiveness of intergroup contact. This neglect may be more likely if governmental anti-stigma programme funders do not wish to make structural changes, for political and/or economic reasons. This is not to deny, however, that interventions at the interpersonal level may not eventually impact on structural discrimination. For example, positive changes in favour of fewer stigmatising articles as a proportion of all newspaper coverage on mental health [92,93] are likely to be in response to changes in public attitudes to mental illness [2], which newspaper staff will detect as their readerships’ responses to articles change.

## 5. Structural Intervention

The starting point for governments wishing to reduce structural discrimination is the adoption of anti-discrimination legislation which applies to mental illness. In the case of US and most European legislation, many people with mental illness are covered within the protected category of people with a disability. For example, in Britain under the Equality Act 2010 [94,95], disability is defined as physical or mental impairment that has a ‘substantial’ and ‘long-term’ negative effect on the ability to do normal daily activities. ‘Long-term’ is defined as lasting, or expected to last, 12 months or more. The definition applies even when the effects on activities are managed by treatment such as medication. Discrimination may be direct in that it targets a person or a group of people with disability due to mental illness; or indirect, in the case of something that puts them at a relative disadvantage. Someone who associates with someone in a protected category and experience this ‘discrimination by association’ is also protected, as is someone wrongly assumed to have a protected characteristic who experiences ‘perception discrimination’. Since the introduction of the Equality Act 2010, clauses in other legislation which were themselves discriminatory have been removed. These excluded people who had been subject to the Mental Health Act from certain roles, including Member of Parliament, school governor, company board member, and member of a jury.

Anti-discrimination legislation applies to employers and service providers. In cases of direct discrimination, the person or people affected must pursue a case themselves, for example, an Employment Tribunal in the case of discrimination by an employer in the UK. However, the pursuit of a discrimination case may be harder for someone currently experiencing a mental illness compared to people covered by most of the other protected categories such as gender, race/ethnicity, or age. Further, in terms of the administration of justice, one issue of concern raised by recent UK research [96] was the significant variation in detail and quality of Employment Tribunal Judgments. It was not unusual for the Employment Appeal Tribunal to observe, in cases where they allowed an appeal or remitted it for a fresh hearing, that ‘in their view the employment tribunal’s fact finding was economical, explanation for decisions were lacking in detail and reasons for particular conclusions or inferences drawn were not fully explained’ [97]. Specific failings by tribunals in relation to people with disability due to mental illness included: failure to establish the effects of the disability; failure to take into account medical evidence submitted by the claimant; and failure to properly consider the complaints that the employer had failed to provide a reasonable adjustment in order that the claimant could carry out their job [96]. Such failings show that the justice system fails in some cases to address structural discrimination when cases are brought, and as a result it perpetuates such discrimination.

Like the criminal justice system, any organisation or person in contact with people with mental illness is both part of the problem of stigma as well as part of the solution. This also applies to people with mental health problems, who can both stigmatise others and confront stigma on the part of others. However, given the usual power differential between those experiencing discrimination and others, it is important for all employers and service provider organisations to review their policies for direct or indirect discrimination. Relatively few cases are brought for indirect discrimination as it affects people who are covered by legislation as disabled due to mental illness, but it should not be assumed that this is rare, rather that it may be relatively difficult to bring such cases.

Funding for mental health care and mental health research is universally relatively low [98,99]. For some types of mental health care in the UK, lack of NHS provision has led to either care provided far from a service users home or care which is often of poorer quality provided outside the NHS, which is also often located remotely [100]. In some former communist countries, the quality of inpatient psychiatric care is in sharp contrast to physical health provision [101] and has custodial aspects also found in some lower income countries. Access to primary care in the UK is not equalised through the use of any routine, active process to identify patients who find it difficult to wait surrounded by others or who need additional time for an appointment. Such adjustments are made to varying extents for patients with severe mental illness as these problems become known, but there is still a stark contrast between how such provisions made for people with mental illness and the extended appointments provided for health reviews for people with learning disabilities, in spite of the similar reduction in life expectancy between these two groups [102,103]. Where the authors work, we have come across two examples. First, some district nurse services have a policy of refusing to visit anyone who is not housebound due to a physical disability; this discriminates against anyone unable to attend for primary health care due to symptoms of mental illness. Second, a mapping exercise of health promotion programmes in four local government areas in London [104], which are run by local government or the voluntary sector as well as by health services, showed that some exclude people with severe mental illness, or impose requirements that they be consistently using prescribed medication or attend with a care coordinator. The latter effectively excludes such people as care coordinators do not generally have time to attend. None of the publicly funded leisure centres identified in this exercise had anyone trained to include people with severe mental illness in health promotion programmes.

## 6. Conclusions

We conclude that considering stigma as a wicked problem shows that attention should be paid to trying to anticipate and then identify unintended consequences of both anti-stigma interventions and research into them. Second, there is value in reformulation of the problem of stigma, for example, to encourage a broader range of interventions than those studied to date, to include structural level interventions in multiple service sectors rather than just health care. Third, we need to better understand how to integrate effective contact into workplaces and educational settings so that it can have a sustained impact. Thus, the success of current anti-stigma programmes should be judged not just on the basis of concurrent evaluation but on the legacy they leave; reducing stigma must become part of the core business of all relevant stakeholders, whether national or local government, health care organisations, professional groups [105], mental health charities, consumer and carer groups. We argue it should also be part of the core business of organisations to whom anti-discrimination legislation applies.
